# Supplementary figures and images for: The Healing Effect of Human Milk Fat Globule-EGF Factor 8 Protein (MFG-E8) in A Rat Model of Parkinson’s Disease
Source: Brain Sci. 2018 Aug 31;8(9):167. doi: 10.3390/brainsci8090167 (PMC6162645; doi:10.3390/brainsci8090167)

**A**

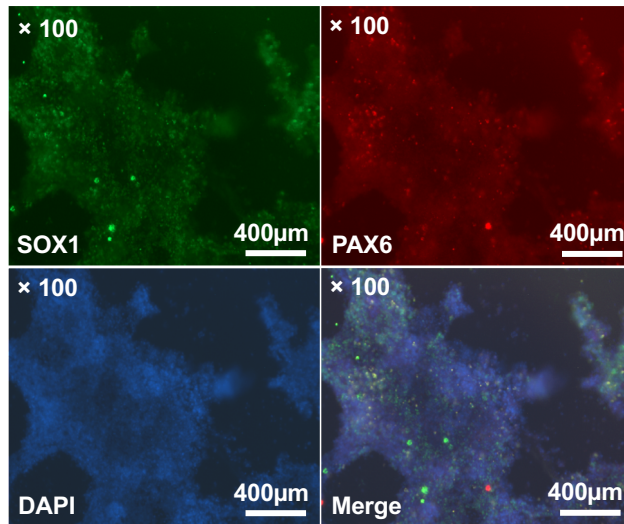

**B**

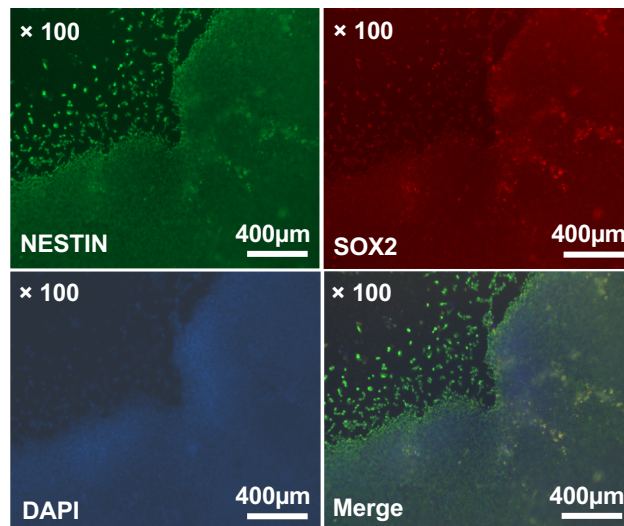

**C**

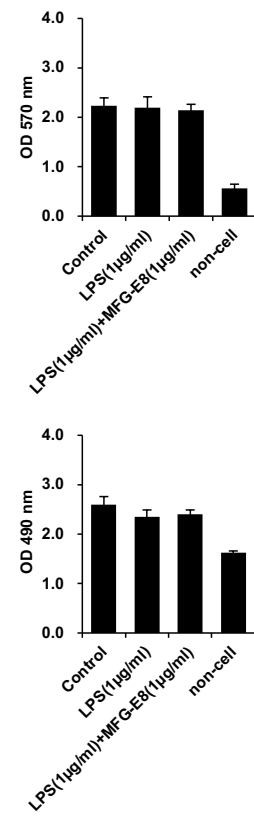

Supplement: Supplementary file 1 [file brainsci-08-00167-s001.pdf]
